# Supplementary material for: Identification of Fungicide Combinations Targeting Plasmopara viticola and Botrytis cinerea Fungicide Resistance Using Machine Learning
Source: Microorganisms. 2023 May 19;11(5):1341. doi: 10.3390/microorganisms11051341 (PMC10221559; doi:10.3390/microorganisms11051341)
Supplement: Supplementary file 1 [file microorganisms-11-01341-s001.zip › microorganisms-2301758-supplementary.pdf]

Supplementary Data

Table S1. Compound Information for all the fungicides.

Compound #	Structure	Name
1	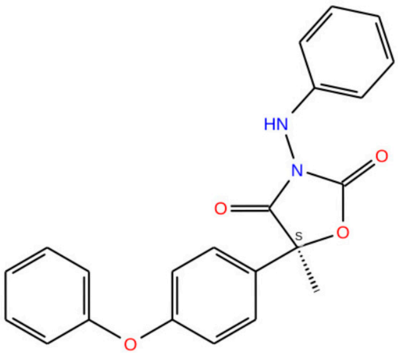	Famoxadone
2	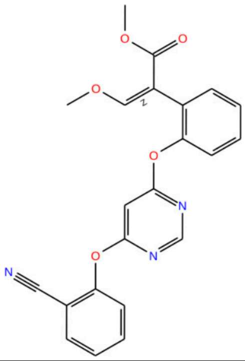	Azoxystrobin
3	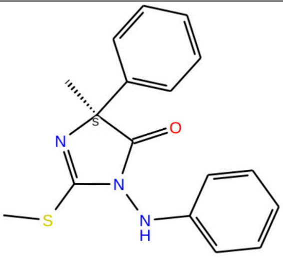	Fenamidone
4	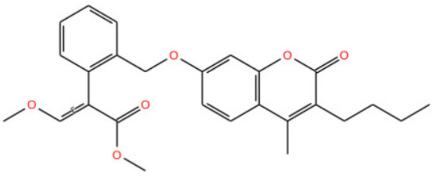	Coumoxystrobin

5	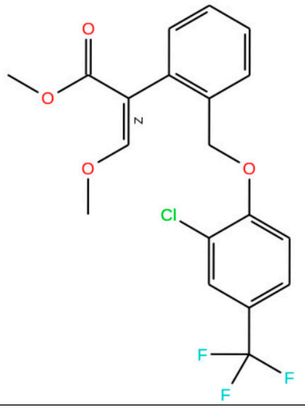	Flufenoxystrobin
6	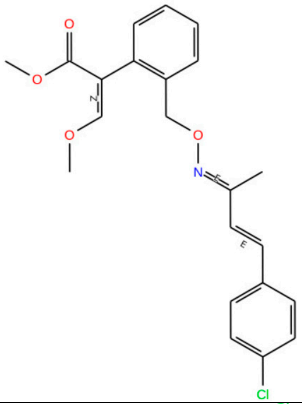	Enoxastrobin
7	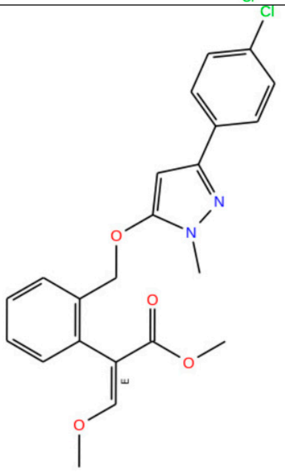	Pyraoxystrobin
8	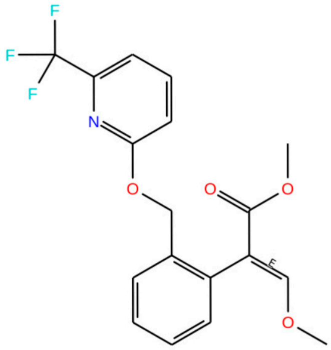	Picoxystrobin

9	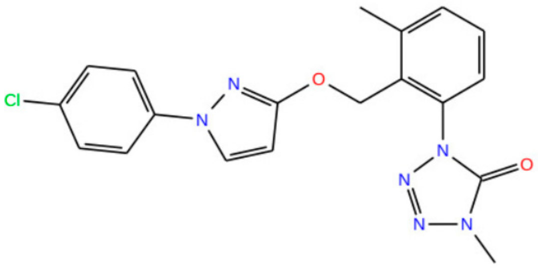	Metyltetraprole
10	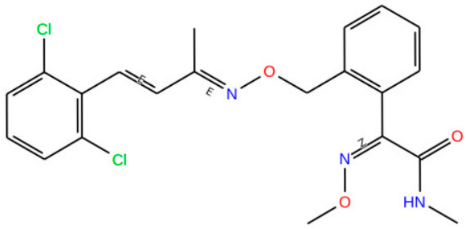	Fenaminstrobin
11	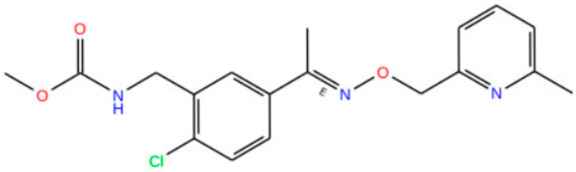	Pyribencarb
12	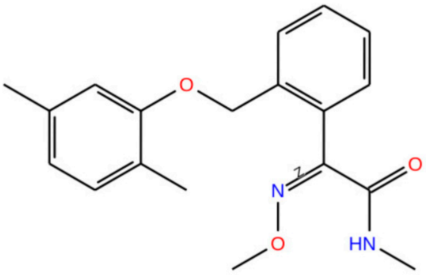	Dimoxystrobin
13	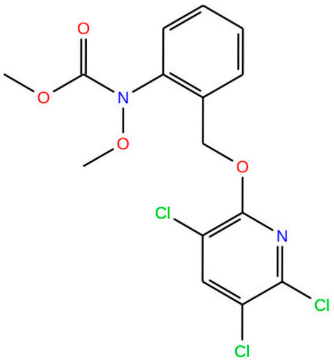	Triclopyricarb

14	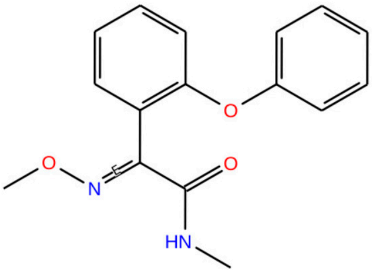	Metominostrobin
15	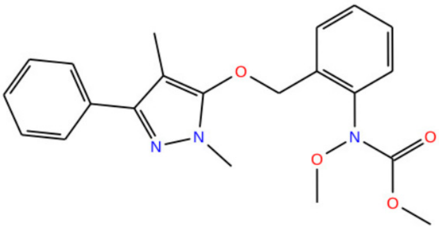	Pyrametostrobin
16	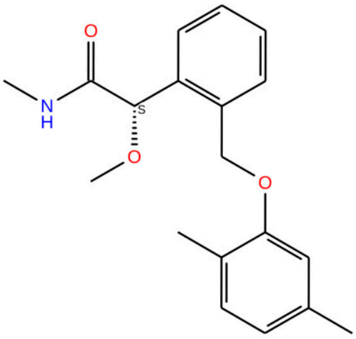	Mandestrobin
17	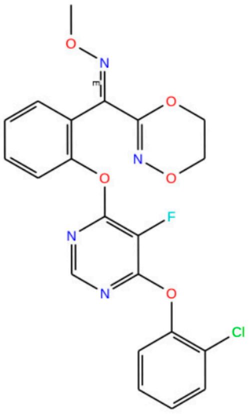	Fluoxastrobin

18	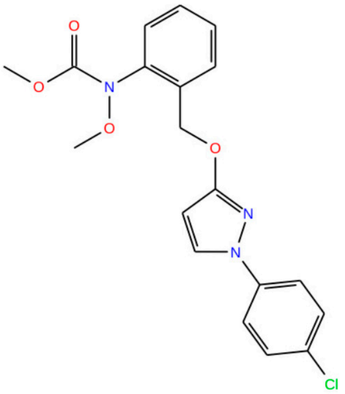	Pyraclostrobin
19	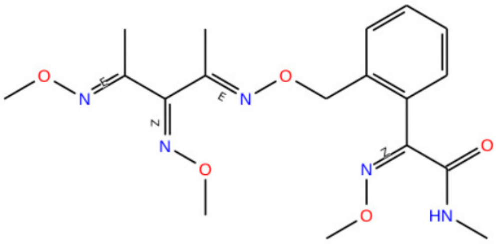	Orysastrobin
20	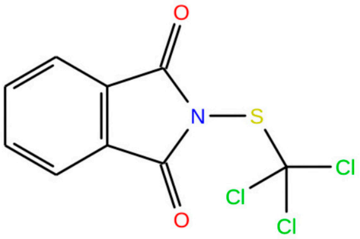	Folpet
21	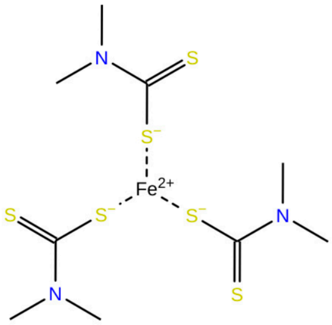	Ferbam
22	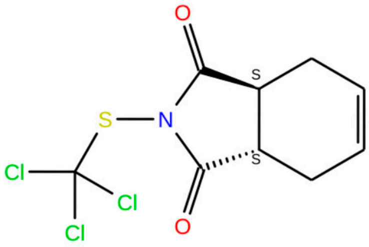	Captan

23	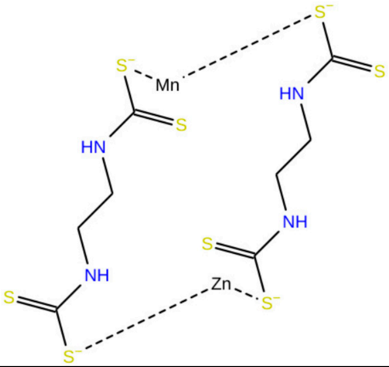	Mancozeb
24	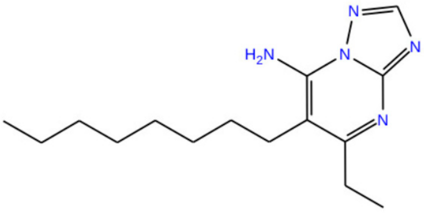	Ametoctradin
25	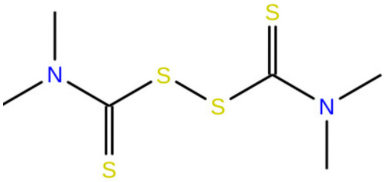	Thiram
26	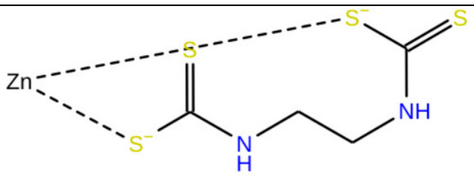	Zineb
27	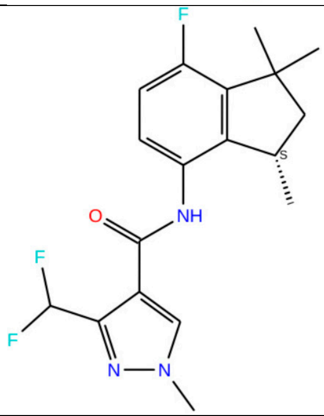	Fluindapyr

28	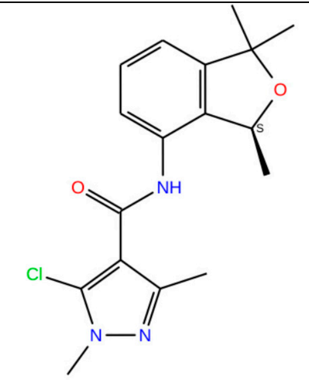	Furametpyr
29	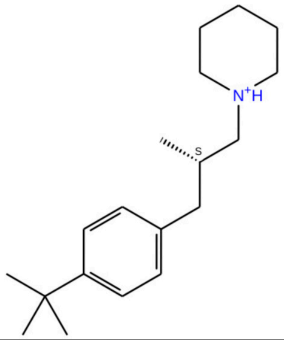	Fenpropidin
30	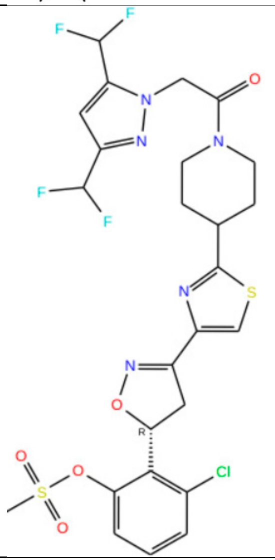	Fluoxapiprolin
31	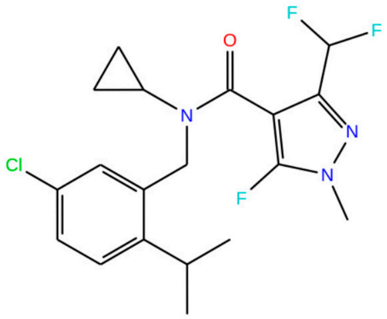	Isoflucypram

32	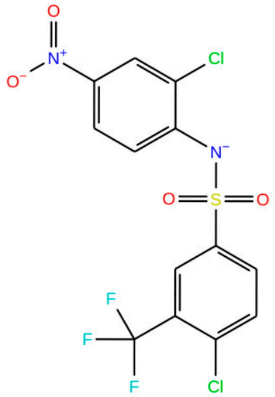	Flusulfamide
33	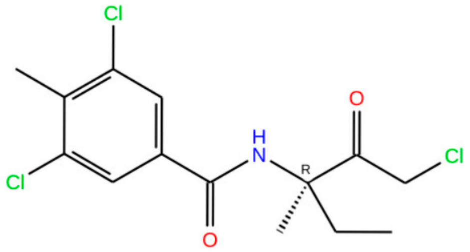	Zoxamide
34	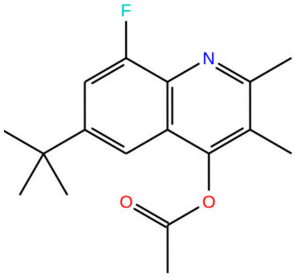	Tebufloquin
35	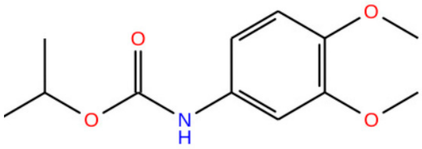	Diethofencarb
36	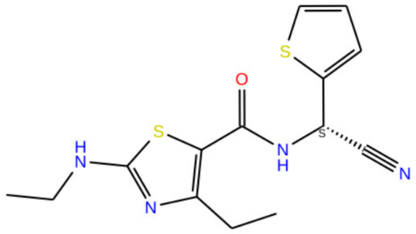	Ethaboxam

37	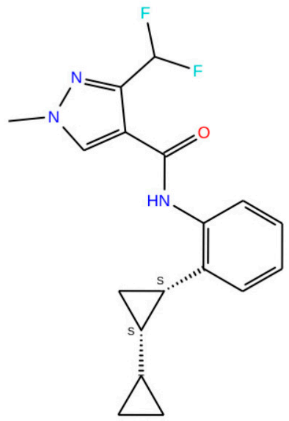	Sedaxane
38	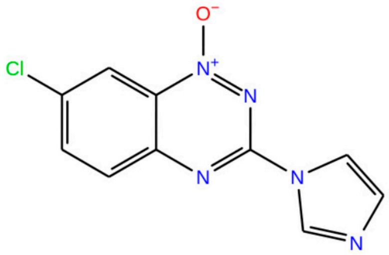	Triazoxide
39	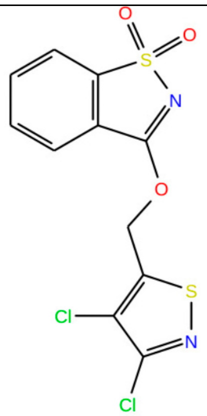	Dichlobentiazox
40	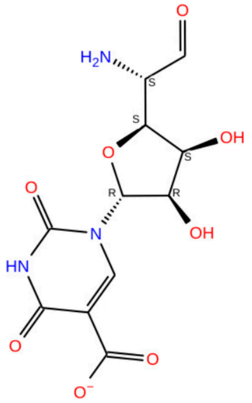	Polyoxin

41	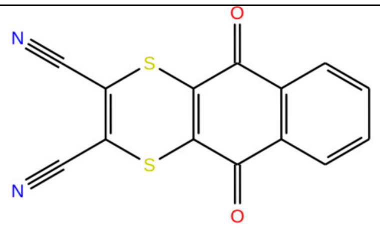	Dithianon
42	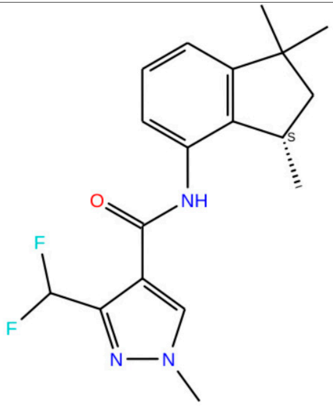	Inpyrfluxam
43	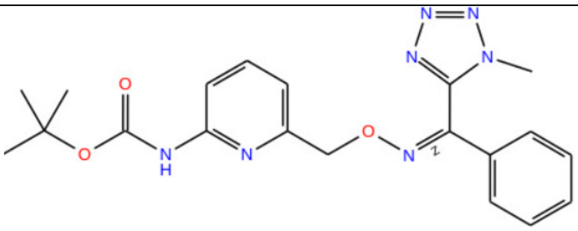	Picarbutrazox
44	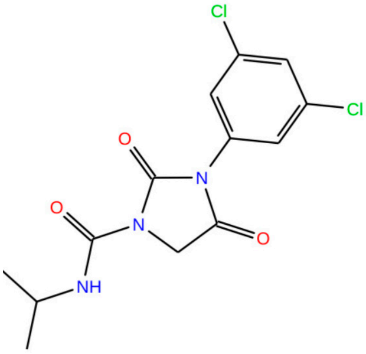	Iprodione
45	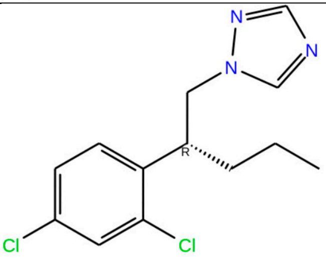	Penconazole

46	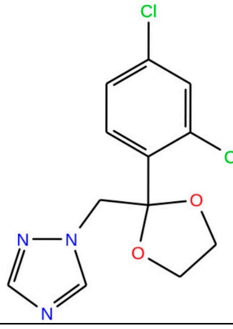	Azaxonazole
47	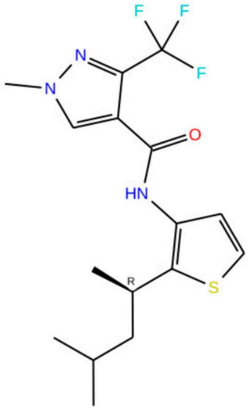	Penthiopyrad
48	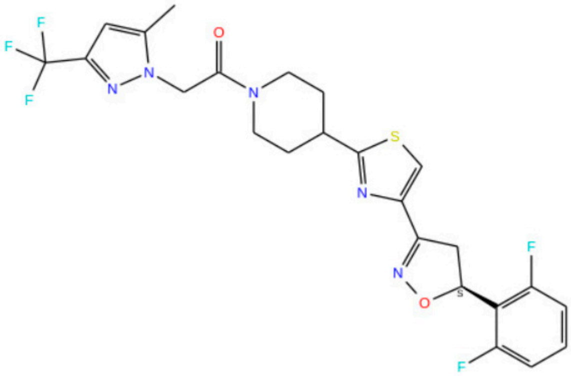	Oxathiaprolin
49	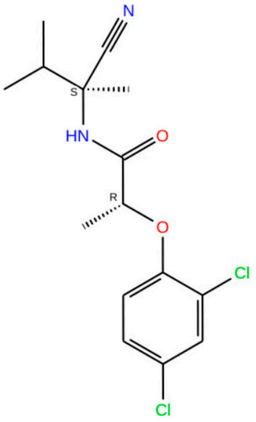	Fenoxanil

50	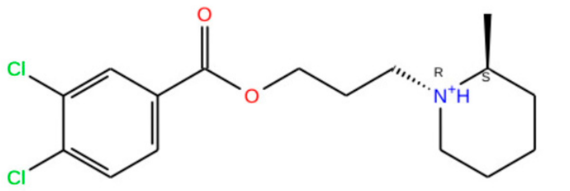	Piperalin
51	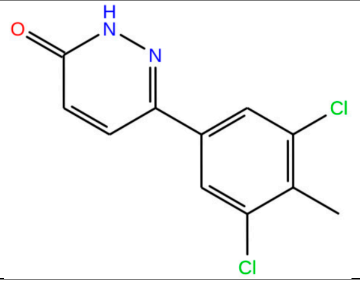	Diclomezine
52	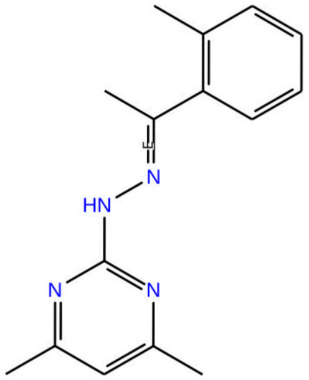	Ferimzone
53	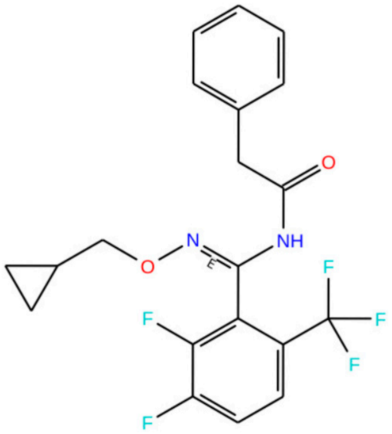	Cyflufenamid

Table S2. Calculated binding affinity (via docking simulations) and predicted binding affinity between 19 selected ligands and G143A-mutated cytochrome b of *Botrytis cinerea* without a validation set.

Fungicide	Calculated Binding Affinity	Predicted Binding Affinity
Furametpyr	-2.705	-7.218
Azaconazole	-5.702	-7.084
Penthiopyrad	-4.203	-6.899
Oxathiapiprolin	-3.960	-6.481
Triazoxide	-2.937	-6.233
Fenpropidin	-4.878	-5.989
Fenoxanil	-4.928	-5.862
Isoflucypram	-5.261	-5.737
Ametoctradin	-6.954	-5.485
Flusulfamide	-5.418	-5.410
Polyoxin	-4.119	-5.132
Diethofencarb	-1.966	-4.710
Tebufloquin	-4.352	-4.359
Mandestrobin	-8.690	-4.224
Picarbutrazox	-0.453	-4.068
Famoxadone	-8.765	-3.443
Iprodione	-4.797	-3.353
Dithianon	-2.515	-3.347
Metominostrobin	-7.417	-2.032

Table S3. Calculated binding affinity (via docking simulations) and predicted binding affinity between 19 selected ligands and G143A-mutated cytochrome b of *Botrytis cinerea* for QSAR model with a validation set.

Fungicide	Calculated Binding Affinity	Predicted Binding Affinity
Penthiopyrad	-4.203	-6.652
Isoflucypram	-5.261	-6.601
Oxathiapiprolin	-3.960	-6.351
Azaconazole	-5.702	-6.137
Furametpyr	-2.705	-6.077
Flusulfamide	-5.418	-5.391
Fenoxanil	-4.928	-5.288
Triazoxide	-2.937	-5.241
Fenpropidin	-4.878	-5.115
Iprodione	-4.797	-4.581
Tebufloquin	-4.352	-4.436
Ametoctradin	-6.954	-4.396
Polyoxin	-4.119	-4.141
Diethofencarb	-1.966	-3.925
Famoxadone	-8.765	-3.726
Picarbutrazox	-0.453	-3.643
Dithianon	-2.515	-3.639
Mandestrobin	-8.690	-3.244
Metominostrobin	-7.417	-1.958

Table S4. Calculated binding affinity (via docking simulations) and predicted binding affinity between 17 selected ligands and G143A-mutated cytochrome b of *Plasmopara viticola* without a validation set.

Fungicide	Calculated Binding Affinity	Predicted Binding Affinity
Fluindapyr	-5.424	-8.091
Furametpyr	-4.667	-6.826
Fenpropidin	-4.410	-6.714
Fluoxapiprolin	-2.857	-6.600
Isoflucypram	-1.367	-6.354
Flusulfamide	-5.110	-5.920
Ametoctradin	-6.299	-5.824
Tebufloquin	0	-5.767
Diethofencarb	-3.406	-5.649
Ethaboxam	-5.662	-4.842
Famoxadone	-6.238	-4.785
Triazoxide	0	-4.617
Mandestrobin	-7.393	-4.611
Polyoxin	-3.621	-4.568
Dithianon	0	-4.306
Dimoxystrobin	-7.548	-4.034
Picarbutrazox	-4.188	-2.413

Table S5. Calculated binding affinity (via docking simulations) and predicted binding affinity between 17 selected ligands and G143A-mutated cytochrome b of *Plasmopara viticola* by using the QSAR model with a validation set.

Fungicide	Calculated Binding Affinity	Predicted Binding Affinity
Fluindapyr	-5.424	-7.845
Furametpyr	-4.667	-6.300
Flusulfamide	-5.110	-6.275
Isoflucypram	-1.367	-6.054
Fenpropidin	-4.410	-5.903
Diethofencarb	-3.406	-5.608
Tebufloquin	0	-5.436
Fluoxapiprolin	-2.857	-5.307
Triazoxide	0	-4.943
Ametoctradin	-6.299	-4.740
Ethaboxam	-5.662	-4.672
Dithianon	0	-4.287
Polyoxin	-3.621	-4.257
Famoxadone	-6.238	-4.117
Mandestrobin	-7.393	-3.989
Dimoxystrobin	-7.548	-3.157
Picarbutrazox	-4.188	-2.351

QSAR Data for Botrytis cinerea

Model Report

Model Code	Score	S.D.	R^2	RMSE	Q^2	Q^2 MW (Null Hypothesis)
pls_19	0.5892	2.1184	0.6378	2.1419	0.6172	-0.0635
kpls_radial_19	0.5595	1.8525	0.7110	2.1152	0.6267	-0.0635
kpls_dendritic_19	0.5593	1.6052	0.7830	2.0310	0.6558	-0.0635
kpls_desc_19	0.5544	1.9419	0.6956	2.1414	0.6174	-0.0635
kpls_linear_19	0.5219	1.6506	0.7706	2.1168	0.6261	-0.0635
kpls_radial_39	0.4979	1.9519	0.6875	2.2512	0.5641	-1.2909
kpls_dendritic_34	0.4899	1.7542	0.7610	2.1877	0.5655	-0.5910
kpls_linear_34	0.4792	1.7329	0.7668	2.2016	0.5600	-0.5910
kpls_radial_16	0.4760	1.9404	0.7073	2.2709	0.5352	-1.2336
kpls_dendritic_44	0.4308	1.4941	0.8236	2.2315	0.5745	0.1264

Figure S1. Top 10-ranked QSAR models without a validation set for (fungicides used in) *Botrytis cinerea*.

b)

Training Set		Test Set	
S.D.	R^2	RMSE	Q^2
1.8525	0.7110	2.1152	0.6267

Optimum number of factors = 1

ID	Set	Y(Obs)	Y(Pred)	Error	Name
1	train	0.0000	-0.6358	-0.6358	Azoxystrobin
2	train	0.0000	-0.0303	-0.0303	Coumoxystrobin
3	train	-4.2340	-1.1916	3.0424	Dimoxystrobin
4	test	0.0000	-1.2766	-1.2766	Enoxastrobin
5	train	-5.9500	-4.2829	1.6671	Fenamidone
6	train	0.0000	-0.1511	-0.1511	Fenaminstrobin
7	train	0.0000	-1.4393	-1.4393	Fluoxastrobin
8	train	0.0000	-3.1365	-3.1365	Metyltetraprole
9	train	0.0000	0.1815	0.1815	Orysastrobin
10	train	-8.2000	-3.5157	4.6843	Picoxystrobin
11	train	-3.4420	-2.6442	0.7978	Pyraclostrobin
12	train	0.0000	-1.5631	-1.5631	pyrametostrobin
13	train	-4.7170	-2.6272	2.0898	Pyraoxystrobin
14	train	-8.4460	-7.8597	0.5863	Pyribencarb
15	test	0.0000	-3.8050	-3.8050	Triclopyricarb
16	train	0.0000	-1.9058	-1.9058	Captan
17	test	-3.1360	-3.2042	-0.0682	Ferbam
18	train	0.0000	-1.5244	-1.5244	Folpet
19	train	0.0000	-2.1791	-2.1791	Mancozeb
20	train	-4.9350	-3.3297	1.6053	Thiram
21	train	-2.4520	-2.1791	0.2729	Zineb
22	train	-7.2440	-7.5121	-0.2681	Zoxamide
23	test	-9.2080	-6.1196	3.0884	Sedaxane
24	test	-8.9120	-7.5089	1.4031	Piperalin
25	train	-6.2170	-7.4936	-1.2766	Inpyrfluxam
26	train	-8.8700	-8.0283	0.8417	Penconazole
27	train	-6.0890	-5.4897	0.5993	Ferimzone
28	test	-7.3720	-5.2805	2.0915	Cyflufenamid
29	test	-6.6920	-4.7438	1.9482	Diclomezine
30	train	-7.2000	-4.9348	2.2652	Dichlobentiazox
31	test	-6.0650	-6.1073	-0.0423	Ethaboxam
32	train	-6.7210	-8.4611	-1.7401	Fluindapyr
33	train	-6.8900	-8.4643	-1.5743	Fluoxapiprolin
34	train	0.0000	-1.2093	-1.2093	Flufenoxystrobin

c)

Training Set		Test Set	
S.D.	R^2	RMSE	Q^2
1.6052	0.7830	2.0310	0.6558

Optimum number of factors = 1

ID	Set	Y(Obs)	Y(Pred)	Error	Name
1	train	0.0000	-0.0943	-0.0943	Azoxystrobin
2	train	0.0000	-0.1075	-0.1075	Coumoxystrobin
3	train	-4.2340	-1.6236	2.6104	Dimoxystrobin
4	test	0.0000	-0.9813	-0.9813	Enoxastrobin
5	train	-5.9500	-5.5197	0.4303	Fenamidone
6	train	0.0000	-0.6974	-0.6974	Fenaminstrobin
7	train	0.0000	0.3008	0.3008	Fluoxastrobin
8	train	0.0000	-2.5778	-2.5778	Metyltetraprole
9	train	0.0000	-0.1490	-0.1490	Orysastrobin
10	train	-8.2000	-4.8191	3.3809	Picoxystrobin
11	train	-3.4420	-2.6495	0.7925	Pyraclostrobin
12	train	0.0000	-0.5525	-0.5525	pyrametostrobin
13	train	-4.7170	-3.2325	1.4845	Pyraoxystrobin
14	train	-8.4460	-8.6584	-0.2124	Pyribencarb
15	test	0.0000	-2.5071	-2.5071	Triclopyricarb
16	train	0.0000	-1.8739	-1.8739	Captan
17	test	-3.1360	-3.2377	-0.1017	Ferbam
18	train	0.0000	-1.4578	-1.4578	Folpet
19	train	0.0000	-2.6136	-2.6136	Mancozeb
20	train	-4.9350	-3.3862	1.5488	Thiram
21	train	-2.4520	-2.6136	-0.1616	Zineb
22	train	-7.2440	-7.9947	-0.7508	Zoxamide
23	test	-9.2080	-6.3464	2.8616	Sedaxane
24	test	-8.9120	-6.1379	2.7741	Piperalin
25	train	-6.2170	-8.4638	-2.2468	Inpyrfluxam
26	train	-8.8700	-7.7345	1.1355	Penconazole
27	train	-6.0890	-5.0672	1.0218	Ferimzone
28	test	-7.3720	-4.3515	3.0205	Cyflufenamid
29	test	-6.6920	-6.2629	0.4291	Diclomezine
30	train	-7.2000	-4.3167	2.8833	Dichlobentiazox
31	test	-6.0650	-5.3245	0.7405	Ethaboxam
32	train	-6.7210	-7.8362	-1.1152	Fluindapyr
33	train	-6.8900	-7.5913	-0.7013	Fluoxapiprolin
34	train	0.0000	-0.2772	-0.2772	Flufenoxystrobin

d)

Training Set		Test Set	
S.D.	R^2	RMSE	Q^2
1.9419	0.6956	2.1414	0.6174

Optimum number of factors = 2

ID	Set	Y(Obs)	Y(Pred)	Error	Name
1	train	0.0000	-1.0399	-1.0399	Azoxystrobin
2	train	0.0000	-0.9394	-0.9394	Coumoxystrobin
3	train	-4.2340	-4.0657	0.1683	Dimoxystrobin
4	test	0.0000	-0.4840	-0.4840	Enoxastrobin
5	train	-5.9500	-3.7535	2.1965	Fenamidone
6	train	0.0000	0.0520	0.0520	Fenaminstrobin
7	train	0.0000	0.5181	0.5181	Fluoxastrobin
8	train	0.0000	-2.2374	-2.2374	Metyltetraprole
9	train	0.0000	-1.7786	-1.7786	Orysastrobin
10	train	-8.2000	-3.7445	4.4555	Picoxystrobin
11	train	-3.4420	-2.0358	1.4062	Pyraclostrobin
12	train	0.0000	-3.1445	-3.1445	pyrametostrobin
13	train	-4.7170	-2.3666	2.3504	Pyraoxystrobin
14	train	-8.4460	-5.0040	3.4420	Pyribencarb
15	test	0.0000	-2.3964	-2.3964	Triclopyricarb
16	train	0.0000	0.2969	0.2969	Captan
17	test	-3.1360	-2.4665	0.6695	Ferbam
18	train	0.0000	0.6486	0.6486	Folpet
19	train	0.0000	-1.1119	-1.1119	Mancozeb
20	train	-4.9350	-4.7803	0.1547	Thiram
21	train	-2.4520	-4.1275	-1.6755	Zineb
22	train	-7.2440	-6.9948	0.2492	Zoxamide
23	test	-9.2080	-7.7220	1.4860	Sedaxane
24	test	-8.9120	-6.0067	2.9053	Piperalin
25	train	-6.2170	-7.8943	-1.6773	Inpyrfluxam
26	train	-8.8700	-8.8327	0.0373	Penconazole
27	train	-6.0890	-8.0451	-1.9561	Ferimzone
28	test	-7.3720	-2.9784	4.3936	Cyflufenamid
29	test	-6.6920	-6.4725	0.2195	Diclomezine
30	train	-7.2000	-5.3815	1.8185	Dichlobentiazox
31	test	-6.0650	-6.5723	-0.5073	Ethaboxam
32	train	-6.7210	-7.4851	-0.7641	Fluindapyr
33	train	-6.8900	-5.8729	1.0171	Fluoxapiprolin
34	train	0.0000	-2.4866	-2.4866	Flufenoxystrobin

e)

Training Set		Test Set	
S.D.	R^2	RMSE	Q^2
1.6506	0.7706	2.1168	0.6261

Optimum number of factors = 1

ID	Set	Y(Obs)	Y(Pred)	Error	Name
1	train	0.0000	-0.5586	-0.5586	Azoxystrobin
2	train	0.0000	-0.0221	-0.0221	Coumoxystrobin
3	train	-4.2340	-1.5967	2.6373	Dimoxystrobin
4	test	0.0000	-0.7957	-0.7957	Enoxastrobin
5	train	-5.9500	-5.5331	0.4169	Fenamidone
6	train	0.0000	-0.5917	-0.5917	Fenaminstrobin
7	train	0.0000	0.0507	0.0507	Fluoxastrobin
8	train	0.0000	-2.6519	-2.6519	Metyltetraprole
9	train	0.0000	-0.1103	-0.1103	Orysastrobin
10	train	-8.2000	-4.7253	3.4747	Picoxystrobin
11	train	-3.4420	-2.5618	0.8802	Pyraclostrobin
12	train	0.0000	-0.3595	-0.3595	pyrametostrobin
13	train	-4.7170	-2.9254	1.7916	Pyraoxystrobin
14	train	-8.4460	-8.3902	0.0558	Pyribencarb
15	test	0.0000	-1.8684	-1.8684	Triclopyricarb
16	train	0.0000	-1.8525	-1.8525	Captan
17	test	-3.1360	-3.0021	0.1339	Ferbam
18	train	0.0000	-1.4263	-1.4263	Folpet
19	train	0.0000	-2.5984	-2.5984	Mancozeb
20	train	-4.9350	-3.3742	1.5608	Thiram
21	train	-2.4520	-2.5984	-0.1464	Zineb
22	train	-7.2440	-7.9683	-0.7243	Zoxamide
23	test	-9.2080	-6.3072	2.9008	Sedaxane
24	test	-8.9120	-5.6680	3.2440	Piperalin
25	train	-6.2170	-8.5667	-2.3497	Inpyrfluxam
26	train	-8.8700	-7.6174	1.2525	Penconazole
27	train	-6.0890	-5.0843	1.0047	Ferimzone
28	test	-7.3720	-4.1967	3.1753	Cyflufenamid
29	test	-6.6920	-6.1556	0.5364	Diclomezine
30	train	-7.2000	-4.2869	2.9131	Dichlobentiazox
31	test	-6.0650	-4.5171	1.5479	Ethaboxam
32	train	-6.7210	-7.9302	-1.2092	Fluindapyr
33	train	-6.8900	-7.8001	-0.9101	Fluoxapiprolin
34	train	0.0000	-0.5271	-0.5271	Flufenoxystrobin

Figure S2. Model reports for a) refer to the main paper, b) kpls_radial_19, c) kpls_dendritic_19, d) kpls_desc_19, and e) kpls_linear_19 models of *Botrytis cinerea* without using validation set.

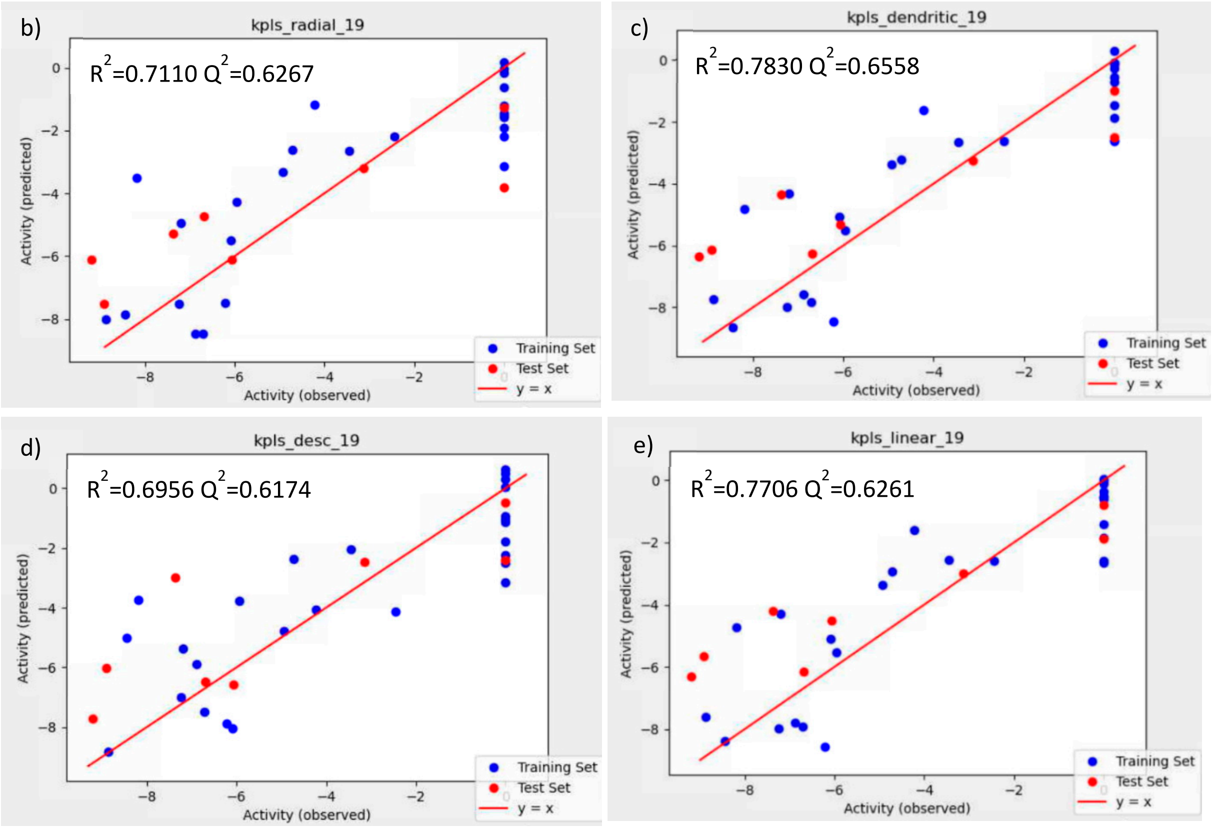

Figure S3. Scatter plot about performance for b) kpls_radial_19, c) kpls_dendritic_19, d) kpls_desc_19, and e) kpls_linear_19 models of *Botrytis cinerea* without using validation set.

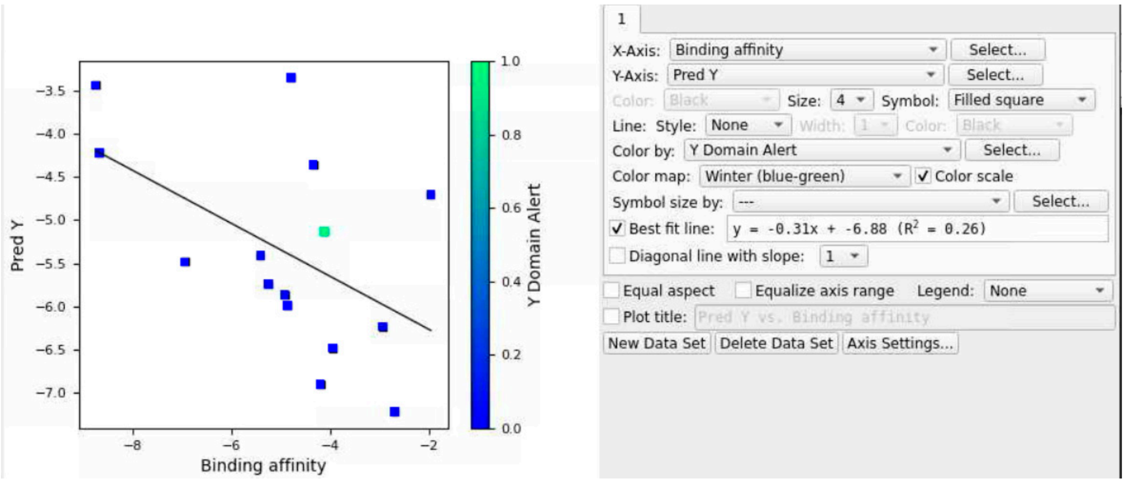

Figure S4. Scatter plot of external validation set after removing four outliers in Figure 10 (c).

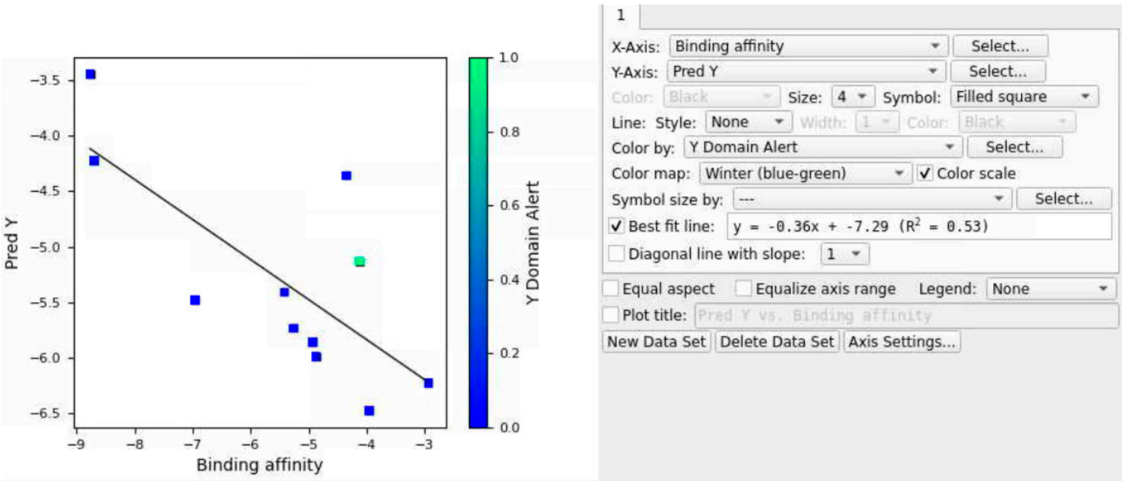

Figure S5. Scatter plot of external validation set after removing four outliers in Figure S4.

Model Report

Model Code	Score	S.D.	R^2	RMSE	Q^2	Q^2 MW (Null Hypothesis)
kpls_molprint2D_39	0.6582	1.8732	0.7081	1.8919	0.6860	0.1725
kpls_radial_8	0.6570	1.8034	0.7316	1.8652	0.6865	-0.5827
kpls_linear_30	0.6218	1.9272	0.7152	1.9482	0.5546	-1.5647
kpls_dendritic_30	0.6068	1.8913	0.7258	1.9626	0.5480	-1.5647
kpls_dendritic_39	0.5888	1.4775	0.8184	1.8814	0.6894	0.1725
kpls_linear_39	0.5818	1.4847	0.8167	1.8971	0.6842	0.1725
kpls_linear_40	0.5717	1.6210	0.7815	1.9602	0.6613	0.1650
kpls_radial_39	0.5714	1.6681	0.7686	1.9765	0.6573	0.1725
kpls_radial_30	0.5322	1.9012	0.7229	2.0992	0.4829	-1.5647
kpls_molprint2D_40	0.5297	1.9253	0.6918	2.1363	0.5977	0.1650

Figure S6. Top 10-ranked QSAR model reports with validation sets for *Botrytis cinerea*.

b)

Training Set		Test Set	
S.D.	R^2	RMSE	Q^2
1.8034	0.7316	1.8652	0.6865

Optimum number of factors = 1

ID	Set	Y(Obs)	Y(Pred)	Error	Name
1	train	0.0000	-0.3619	-0.3619	Azoxystrobin
2	train	0.0000	0.1319	0.1319	Coumoxystrobin
3	train	-4.2340	-1.1572	3.0768	Dimoxystrobin
4	train	0.0000	0.4464	0.4464	Enoxastrobin
5	test	-5.9500	-3.6137	2.3363	Fenamidone
6	test	0.0000	-0.4535	-0.4535	Fenaminstrobin
7	train	0.0000	-2.1305	-2.1305	Fluoxastrobin
8	train	0.0000	-2.9093	-2.9093	Metyltetraprole
9	train	0.0000	0.4589	0.4589	Orysastrobin
10	test	-3.4420	-1.4727	1.9693	Pyraclostrobin
11	train	0.0000	-1.6323	-1.6323	pyrametostrobin
12	train	-4.7170	-1.8294	2.8876	Pyraoxystrobin
13	test	0.0000	-2.5428	-2.5428	Triclopyricarb
14	train	0.0000	-2.2462	-2.2462	Captan
15	train	-3.1360	-3.0359	0.1001	Ferbam
16	train	0.0000	-1.9878	-1.9878	Folpet
17	train	0.0000	-2.2509	-2.2509	Mancozeb
18	train	-4.9350	-3.2755	1.6595	Thiram
19	train	-2.4520	-2.2509	0.2011	Zineb
20	train	-7.2440	-7.2460	-0.0020	Zoxamide
21	train	-9.2080	-7.9810	1.2270	Sedaxane
22	test	-8.9120	-6.2117	2.7003	Piperalin
23	train	-6.2170	-7.6173	-1.4003	Inpyrfluxam
24	train	-8.8700	-8.4120	0.4580	Penconazole
25	train	-6.0890	-3.8953	2.1937	Ferimzone
26	train	-7.3720	-6.5400	0.8320	Cyflufenamid
27	train	-6.6920	-6.5711	0.1209	Diclomezine
28	train	-7.2000	-4.3108	2.8892	Dichlobentiazox
29	test	-6.0650	-4.8418	1.2232	Ethaboxam
30	train	-6.7210	-8.4829	-1.7619	Fluindapyr
31	test	-6.8900	-6.2315	0.6585	Fluoxapiprolin
32	test	0.0000	-1.6132	-1.6132	Flufenoxystrobin

c)

Training Set		Test Set	
S.D.	R^2	RMSE	Q^2
1.9272	0.7152	1.9482	0.5546

Optimum number of factors = 1

ID	Set	Y(Obs)	Y(Pred)	Error	Name
1	train	0.0000	-0.8721	-0.8721	Azoxystrobin
2	train	0.0000	0.1781	0.1781	Coumoxystrobin
3	train	-4.2340	-0.9975	3.2365	Dimoxystrobin
4	test	0.0000	-0.8473	-0.8473	Enoxastrobin
5	test	-5.9500	-3.8890	2.0610	Fenamidone
6	train	0.0000	-0.4923	-0.4923	Fenaminstrobin
7	train	0.0000	-0.6838	-0.6838	Fluoxastrobin
8	train	0.0000	-1.8774	-1.8774	Metyltetraprole
9	train	0.0000	-0.5887	-0.5887	Orysastrobin
10	test	-3.4420	-1.4680	1.9740	Pyraclostrobin
11	train	0.0000	-1.0506	-1.0506	pyrametostrobin
12	train	-4.7170	-1.7187	2.9983	Pyraoxystrobin
13	test	0.0000	-1.5683	-1.5683	Triclopyricarb
14	train	0.0000	-3.6663	-3.6663	Captan
15	test	-3.1360	-3.8378	-0.7018	Ferbam
16	test	0.0000	-3.4565	-3.4565	Folpet
17	train	0.0000	-3.6698	-3.6698	Mancozeb
18	train	-4.9350	-3.8476	1.0874	Thiram
19	train	-2.4520	-3.6698	-1.2178	Zineb
20	test	-7.2440	-5.4511	1.7929	Zoxamide
21	train	-9.2080	-9.0403	0.1677	Sedaxane
22	train	-8.9120	-7.2627	1.6493	Piperalin
23	train	-6.2170	-7.9913	-1.7743	Inpyrfluxam
24	train	-8.8700	-8.1030	0.7670	Penconazole
25	train	-6.0890	-4.0332	2.0558	Ferimzone
26	train	-7.3720	-6.5853	0.7867	Cyflufenamid
27	train	-6.6920	-5.4836	1.2084	Diclomezine
28	train	-7.2000	-3.9691	3.2309	Dichlobentiazox
29	train	-6.0650	-5.8516	0.2134	Ethaboxam
30	train	-6.7210	-8.4304	-1.7094	Fluindapyr
31	test	-6.8900	-5.0497	1.8403	Fluoxapiprolin
32	train	0.0000	0.0230	0.0230	Flufenoxystrobin

d)

Training Set		Test Set	
S.D.	R^2	RMSE	Q^2
1.8913	0.7258	1.9626	0.5480

Optimum number of factors = 1

ID	Set	Y(Obs)	Y(Pred)	Error	Name
1	train	0.0000	-0.5673	-0.5673	Azoxystrobin
2	train	0.0000	0.2340	0.2340	Coumoxystrobin
3	train	-4.2340	-1.1062	3.1278	Dimoxystrobin
4	test	0.0000	-0.7538	-0.7538	Enoxastrobin
5	test	-5.9500	-3.9903	1.9597	Fenamidone
6	train	0.0000	-0.5506	-0.5506	Fenaminstrobin
7	train	0.0000	-0.5588	-0.5588	Fluoxastrobin
8	train	0.0000	-1.7685	-1.7685	Metyltetraprole
9	train	0.0000	-0.6863	-0.6863	Orysastrobin
10	test	-3.4420	-1.4775	1.9645	Pyraclostrobin
11	train	0.0000	-1.1330	-1.1330	pyrametostrobin
12	train	-4.7170	-1.9138	2.8032	Pyraoxystrobin
13	test	0.0000	-1.7893	-1.7893	Triclopyricarb
14	train	0.0000	-3.6356	-3.6356	Captan
15	test	-3.1360	-3.8285	-0.6925	Ferbam
16	test	0.0000	-3.4606	-3.4606	Folpet
17	train	0.0000	-3.6513	-3.6513	Mancozeb
18	train	-4.9350	-3.8358	1.0992	Thiram
19	train	-2.4520	-3.6513	-1.1993	Zineb
20	test	-7.2440	-5.4857	1.7583	Zoxamide
21	train	-9.2080	-9.0287	0.1793	Sedaxane
22	train	-8.9120	-7.2815	1.6305	Piperalin
23	train	-6.2170	-8.0864	-1.8694	Inpyrfluxam
24	train	-8.8700	-8.0876	0.7824	Penconazole
25	train	-6.0890	-4.0518	2.0372	Ferimzone
26	train	-7.3720	-6.6783	0.6937	Cyflufenamid
27	train	-6.6920	-5.5091	1.1829	Diclomezine
28	train	-7.2000	-3.9972	3.2028	Dichlobentiazox
29	train	-6.0650	-5.8889	0.1761	Ethaboxam
30	train	-6.7210	-8.4055	-1.6845	Fluindapyr
31	test	-6.8900	-4.9407	1.9493	Fluoxapiprolin
32	train	0.0000	0.1555	0.1555	Flufenoxystrobin

e)

Training Set		Test Set	
S.D.	R^2	RMSE	Q^2
1.4775	0.8184	1.8814	0.6894

Optimum number of factors = 1

ID	Set	Y(Obs)	Y(Pred)	Error	Name
1	train	0.0000	0.1536	0.1536	Azoxystrobin
2	train	0.0000	-0.5037	-0.5037	Coumoxystrobin
3	train	-4.2340	-2.1713	2.0627	Dimoxystrobin
4	test	0.0000	-1.3947	-1.3947	Enoxastrobin
5	train	-5.9500	-4.5512	1.3988	Fenamidone
6	train	0.0000	-1.4053	-1.4053	Fenaminstrobin
7	train	0.0000	-0.6754	-0.6754	Fluoxastrobin
8	train	0.0000	-1.3153	-1.3153	Metyltetraprole
9	test	0.0000	-1.6255	-1.6255	Orysastrobin
10	train	-3.4420	-0.7807	2.6613	Pyraclostrobin
11	train	0.0000	-0.1521	-0.1521	pyrametostrobin
12	train	-4.7170	-2.3859	2.3311	Pyraoxystrobin
13	train	0.0000	-0.5293	-0.5293	Triclopyricarb
14	train	0.0000	-2.0738	-2.0738	Captan
15	test	-3.1360	-2.3349	0.8011	Ferbam
16	train	0.0000	-1.5521	-1.5521	Folpet
17	train	0.0000	-2.3314	-2.3314	Mancozeb
18	test	-4.9350	-2.2346	2.7004	Thiram
19	train	-2.4520	-2.3314	0.1206	Zineb
20	train	-7.2440	-7.7730	-0.5290	Zoxamide
21	test	-9.2080	-6.0367	3.1713	Sedaxane
22	train	-8.9120	-9.3382	-0.4262	Piperalin
23	train	-6.2170	-7.1219	-0.9049	Inpyrfluxam
24	train	-8.8700	-9.3681	-0.4981	Penconazole
25	train	-6.0890	-4.2110	1.8780	Ferimzone
26	train	-7.3720	-6.9636	0.4084	Cyflufenamid
27	test	-6.6920	-5.5961	1.0959	Diclomezine
28	train	-7.2000	-4.6213	2.5787	Dichlobentiazox
29	train	-6.0650	-6.0380	0.0270	Ethaboxam
30	test	-6.7210	-7.0542	-0.3332	Fluindapyr
31	train	-6.8900	-7.6135	-0.7235	Fluoxapiprolin
32	test	0.0000	-2.1040	-2.1040	Flufenoxystrobin

Figure S7. Model reports for a) refer to the main paper, b) kpls_radial_19, c) kpls_dendritic_19, d) kpls_desc_19 and e) kpls_linear_19 models of *Botrytis cinerea* using validation set.

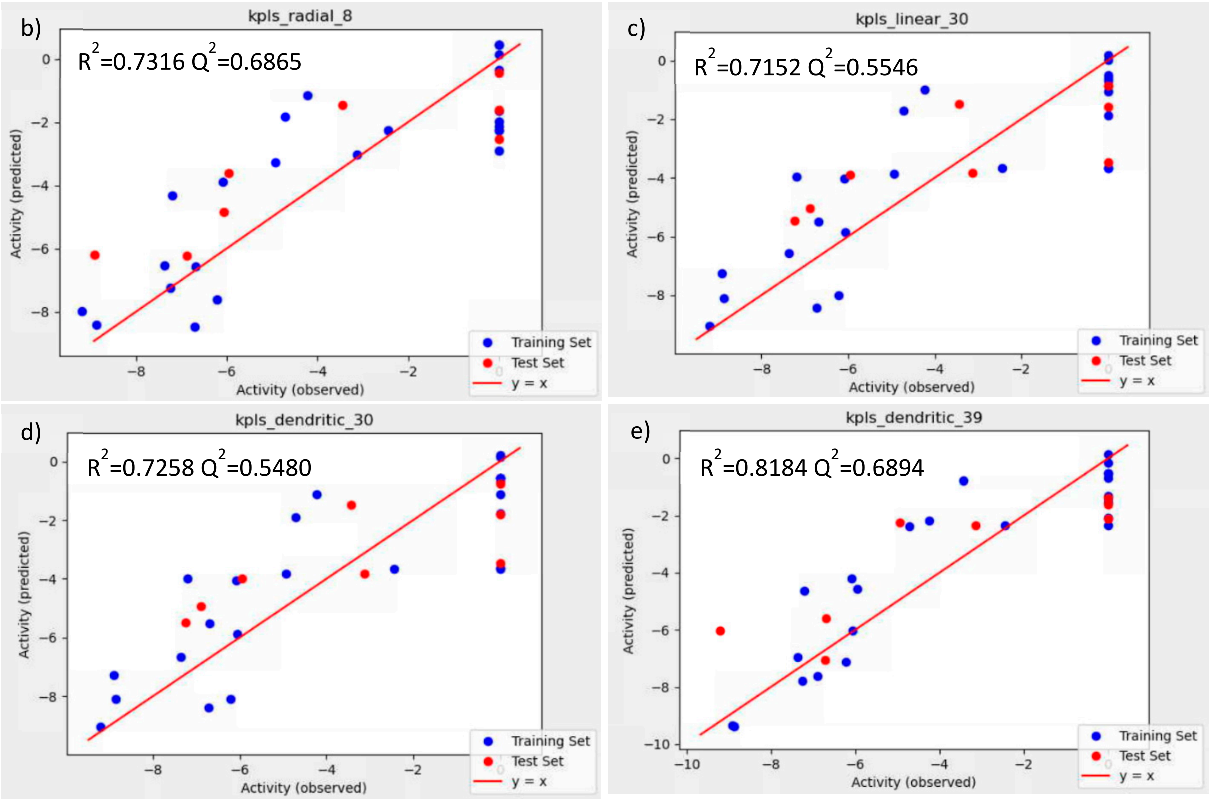

Figure S8. Scatter plot about performance for a) refer to the main paper, b) kpls_radial_8, c) kpls_linear_30, d) kpls_dendritic_30 and e) kpls_dendritic_39 models of *Botrytis cinerea* using validation set.

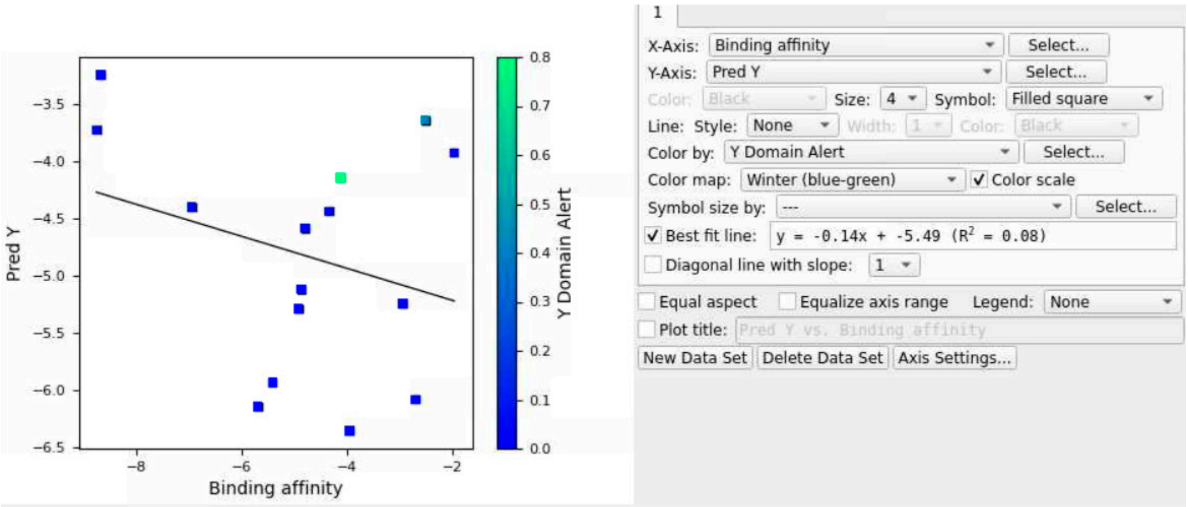

Figure S9. Scatter plot of external validation set after removing four outliers in Figure 11 (c).

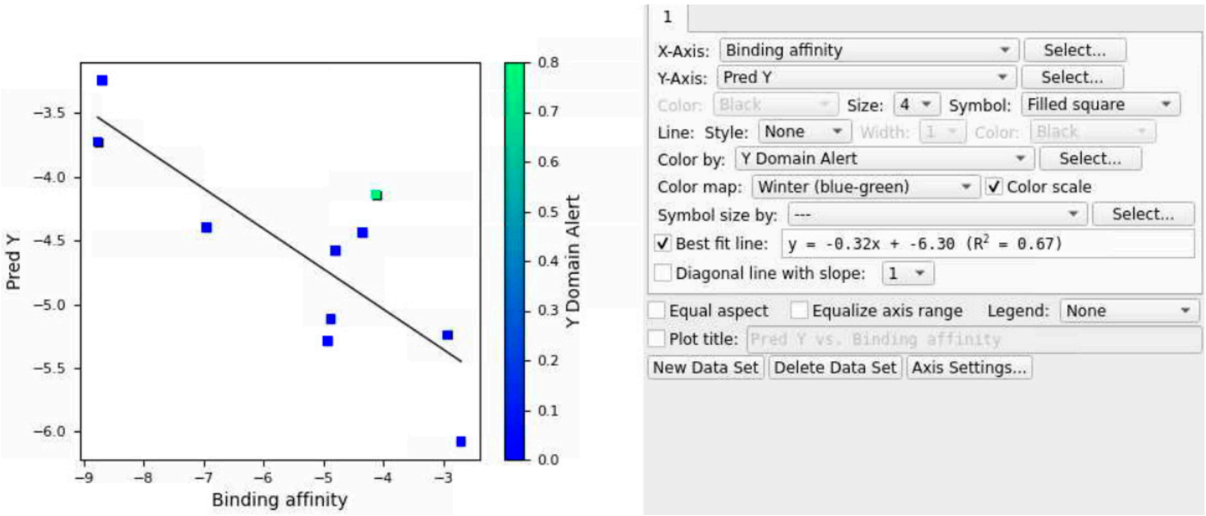

Figure S10. Scatter plot of external validation set after removing five outliers in Figure S9.

QSAR data for Plasmopara vitícola:

Model Report

Model Code	Score	S.D.	R^2	RMSE	Q^2	Q^2 MW (Null Hypothesis)
kpls_desc_2	0.7116	1.7498	0.7032	1.5615	0.7350	0.0226
kpls_radial_24	0.6806	1.5607	0.7489	1.6354	0.7269	0.1147
kpls_linear_22	0.6640	1.4173	0.7931	1.6099	0.7343	0.1112
kpls_radial_22	0.6579	1.5606	0.7491	1.6732	0.7130	0.1112
pls_2	0.6420	1.9451	0.6333	1.7702	0.6594	0.0226
kpls_radial_45	0.6271	1.6222	0.7302	1.7455	0.6800	0.1223
kpls_dendritic_2	0.5756	1.5463	0.7586	1.7997	0.6480	0.0226
kpls_dendritic_22	0.5505	1.4107	0.7950	1.8023	0.6671	0.1112
kpls_linear_47	0.5471	1.4681	0.7821	1.8221	0.6422	0.0948
kpls_radial_47	0.5454	1.6214	0.7342	1.8747	0.6212	0.0948

Figure S11. Top 10-ranked QSAR model reports without validation set for *Plasmopara viticola*.

b)

Training Set		Test Set	
S.D.	R^2	RMSE	Q^2
1.5607	0.7489	1.6354	0.7269

Optimum number of factors = 1

ID	Set	Y(Obs)	Y(Pred)	Error	Name
1	train	0.0000	-0.5844	-0.5844	Azoxystrobin
2	train	-6.1770	-3.4788	2.6982	Coumoxystrobin
3	test	0.0000	-2.8770	-2.8770	Enoxastrobin
4	train	-6.3730	-5.7930	0.5800	Fenamidone
5	train	-7.4660	-4.4231	3.0429	Fenaminstrobin
6	train	0.0000	-0.6105	-0.6105	Fluoxastrobin
7	train	0.0000	-2.2474	-2.2474	Metyltetraprole
8	train	0.0000	-1.7294	-1.7294	Orysastrobin
9	test	0.0000	-2.2333	-2.2333	Picoxystrobin
10	test	0.0000	-0.7546	-0.7546	Pyraclostrobin
11	train	0.0000	0.5119	0.5119	pyrametostrobin
12	train	0.0000	0.1642	0.1642	Pyraoxystrobin
13	train	-5.8440	-4.8203	1.0237	Pyribencarb
14	train	0.0000	-1.0233	-1.0233	Triclopyricarb
15	train	0.0000	-3.6030	-3.6030	Captan
16	test	-3.0380	-3.7681	-0.7301	Ferbam
17	test	-5.8910	-4.3530	1.5380	Folpet
18	train	-1.8780	-2.6717	-0.7937	Mancozeb
19	train	-4.2850	-3.6894	0.5956	Thiram
20	train	-2.4080	-2.6717	-0.2637	Zineb
21	test	-6.5560	-6.0276	0.5284	Zoxamide
22	train	-6.5160	-5.9017	0.6143	Sedaxane
23	train	-6.8510	-7.9132	-1.0622	Piperalin
24	train	-6.4860	-7.1236	-0.6376	Inpyrfluxam
25	train	-7.2910	-8.1414	-0.8504	Penconazole
26	test	-6.4640	-4.2112	2.2528	Ferimzone
27	test	-7.5040	-6.3544	1.1496	Cyflufenamid
28	train	-6.5290	-5.8069	0.7221	Diclomezine
29	train	-6.4320	-4.6564	1.7756	Dichlobentiazox
30	train	-6.2790	-4.2969	1.9821	Flufenoxystrobin
31	train	-7.2240	-7.1865	0.0375	Azaconazole
32	train	-6.1090	-7.5367	-1.4277	Fenoxanil
33	train	-7.2400	-6.3321	0.9079	Iprodione
34	test	-7.2910	-6.4770	0.8140	Penthiopyrad
35	train	-6.6800	-8.2834	-1.6034	Oxathiapiprolin
36	train	-5.0670	-3.2863	1.7807	Metominostrobin

c)

Training Set		Test Set	
S.D.	R^2	RMSE	Q^2
1.4173	0.7931	1.6099	0.7343

Optimum number of factors = 1

ID	Set	Y(Obs)	Y(Pred)	Error	Name
1	train	0.0000	-0.7339	-0.7339	Azoxystrobin
2	train	-6.1770	-4.4838	1.6932	Coumoxystrobin
3	test	0.0000	-2.1268	-2.1268	Enoxastrobin
4	test	-6.3730	-4.1793	2.1937	Fenamidone
5	train	-7.4660	-4.8284	2.6376	Fenaminstrobin
6	train	0.0000	-1.2089	-1.2089	Fluoxastrobin
7	train	0.0000	-0.4627	-0.4627	Metyltetraprole
8	train	0.0000	-2.2218	-2.2218	Orysastrobin
9	test	0.0000	-1.8150	-1.8150	Picoxystrobin
10	train	0.0000	1.1313	1.1313	Pyraclostrobin
11	test	0.0000	-1.5249	-1.5249	pyrametostrobin
12	train	0.0000	-0.0475	-0.0475	Pyraoxystrobin
13	train	-5.8440	-5.2417	0.6023	Pyribencarb
14	train	0.0000	-0.7802	-0.7802	Triclopyricarb
15	train	0.0000	-2.9325	-2.9325	Captan
16	train	-3.0380	-2.8959	0.1421	Ferbam
17	train	-5.8910	-4.2676	1.6234	Folpet
18	train	-1.8780	-2.8687	-0.9907	Mancozeb
19	train	-4.2850	-3.1050	1.1800	Thiram
20	train	-2.4080	-2.8687	-0.4607	Zineb
21	train	-6.5560	-8.3395	-1.7835	Zoxamide
22	train	-6.5160	-6.3522	0.1638	Sedaxane
23	train	-6.8510	-7.6051	-0.7541	Piperalin
24	train	-6.4860	-7.7373	-1.2513	Inpyrfluxam
25	train	-7.2910	-8.1507	-0.8597	Penconazole
26	train	-6.4640	-5.1089	1.3551	Ferimzone
27	test	-7.5040	-5.8253	1.6787	Cyflufenamid
28	test	-6.5290	-6.0916	0.4374	Diclomezine
29	train	-6.4320	-4.5006	1.9314	Dichlobentiazox
30	train	-6.2790	-4.6304	1.6486	Flufenoxystrobin
31	train	-7.2240	-7.6823	-0.4583	Azaconazole
32	test	-6.1090	-5.8568	0.2522	Fenoxanil
33	train	-7.2400	-5.7677	1.4723	Iprodione
34	test	-7.2910	-6.3812	0.9098	Penthiopyrad
35	train	-6.6800	-7.3150	-0.6350	Oxathiapiprolin
36	test	-5.0670	-2.9524	2.1146	Metominostrobin

d)

Training Set		Test Set	
S.D.	R^2	RMSE	Q^2
1.5606	0.7491	1.6732	0.7130

Optimum number of factors = 1

ID	Set	Y(Obs)	Y(Pred)	Error	Name
1	train	0.0000	-0.8496	-0.8496	Azoxystrobin
2	train	-6.1770	-3.5091	2.6679	Coumoxystrobin
3	test	0.0000	-2.1704	-2.1704	Enoxastrobin
4	test	-6.3730	-4.6175	1.7555	Fenamidone
5	train	-7.4660	-4.5114	2.9546	Fenaminstrobin
6	train	0.0000	-1.0117	-1.0117	Fluoxastrobin
7	train	0.0000	-1.1525	-1.1525	Metyltetraprole
8	train	0.0000	-1.8835	-1.8835	Orysastrobin
9	test	0.0000	-2.4794	-2.4794	Picoxystrobin
10	train	0.0000	0.9881	0.9881	Pyraclostrobin
11	test	0.0000	-0.8767	-0.8767	pyrametostrobin
12	train	0.0000	-0.5614	-0.5614	Pyraoxystrobin
13	train	-5.8440	-5.7118	0.1322	Pyribencarb
14	train	0.0000	-0.9616	-0.9616	Triclopyricarb
15	train	0.0000	-3.5025	-3.5025	Captan
16	train	-3.0380	-3.1369	-0.0989	Ferbam
17	train	-5.8910	-4.5472	1.3438	Folpet
18	train	-1.8780	-2.5501	-0.6721	Mancozeb
19	train	-4.2850	-3.4991	0.7859	Thiram
20	train	-2.4080	-2.5501	-0.1421	Zineb
21	train	-6.5560	-7.6263	-1.0703	Zoxamide
22	train	-6.5160	-5.9241	0.5919	Sedaxane
23	train	-6.8510	-8.4378	-1.5868	Piperalin
24	train	-6.4860	-7.2996	-0.8136	Inpyrfluxam
25	train	-7.2910	-7.6751	-0.3841	Penconazole
26	train	-6.4640	-5.3358	1.1282	Ferimzone
27	test	-7.5040	-6.8313	0.6727	Cyflufenamid
28	test	-6.5290	-5.2323	1.2967	Diclomezine
29	train	-6.4320	-4.7903	1.6417	Dichlobentiazox
30	train	-6.2790	-3.7493	2.5297	Flufenoxystrobin
31	train	-7.2240	-6.4769	0.7471	Azaconazole
32	test	-6.1090	-6.1862	-0.0772	Fenoxanil
33	train	-7.2400	-6.0813	1.1587	Iprodione
34	test	-7.2910	-6.6695	0.6215	Penthiopyrad
35	train	-6.6800	-8.6592	-1.9792	Oxathiapiprolin
36	test	-5.0670	-2.2455	2.8215	Metominostrobin

e)

Training Set		Test Set	
S.D.	R^2	RMSE	Q^2
1.9451	0.6333	1.7702	0.6594

Optimum number of factors = 2

ID	Set	Y(Obs)	Y(Pred)	Error	Name
1	test	0.0000	-1.1991	-1.1991	Azoxystrobin
2	train	-6.1770	-3.5475	2.6295	Coumoxystrobin
3	train	0.0000	-0.3819	-0.3819	Enoxastrobin
4	train	-6.3730	-5.5131	0.8599	Fenamidone
5	train	-7.4660	-2.5173	4.9487	Fenaminstrobin
6	train	0.0000	0.5590	0.5590	Fluoxastrobin
7	test	0.0000	-2.3849	-2.3849	Metyltetraprole
8	train	0.0000	-1.7280	-1.7280	Orysastrobin
9	train	0.0000	-2.9018	-2.9018	Picoxystrobin
10	train	0.0000	-1.7005	-1.7005	Pyraclostrobin
11	test	0.0000	-2.7162	-2.7162	pyrametostrobin
12	train	0.0000	-1.4375	-1.4375	Pyraoxystrobin
13	test	-5.8440	-4.0998	1.7442	Pyribencarb
14	train	0.0000	-0.8362	-0.8362	Triclopyricarb
15	train	0.0000	-3.1193	-3.1193	Captan
16	test	-3.0380	-1.3272	1.7108	Ferbam
17	train	-5.8910	-4.6982	1.1928	Folpet
18	train	-1.8780	-2.4618	-0.5838	Mancozeb
19	train	-4.2850	-4.9067	-0.6217	Thiram
20	train	-2.4080	-4.1496	-1.7416	Zineb
21	train	-6.5560	-5.8842	0.6718	Zoxamide
22	train	-6.5160	-7.7344	-1.2184	Sedaxane
23	train	-6.8510	-6.8590	-0.0080	Piperalin
24	train	-6.4860	-8.5448	-2.0588	Inpyrfluxam
25	train	-7.2910	-7.1589	0.1321	Penconazole
26	test	-6.4640	-4.7508	1.7132	Ferimzone
27	train	-7.5040	-6.0471	1.4569	Cyflufenamid
28	test	-6.5290	-5.9267	0.6023	Diclomezine
29	train	-6.4320	-3.3914	3.0406	Dichlobentiazox
30	train	-6.2790	-3.4886	2.7904	Flufenoxystrobin
31	train	-7.2240	-6.9467	0.2773	Azaconazole
32	train	-6.1090	-6.8805	-0.7715	Fenoxanil
33	train	-7.2400	-6.8021	0.4379	Iprodione
34	test	-7.2910	-6.2663	1.0247	Penthiopyrad
35	test	-6.6800	-8.5191	-1.8391	Oxathiapiprolin
36	train	-5.0670	-4.9548	0.1122	Metominostrobin

Figure S12. Model reports for a) refer to the main paper, b) kpls_radial_24, c) kpls_linear_22, d) kpls_radial_22, and e) pls_2 models of *Plasmopara viticola* without using validation set.

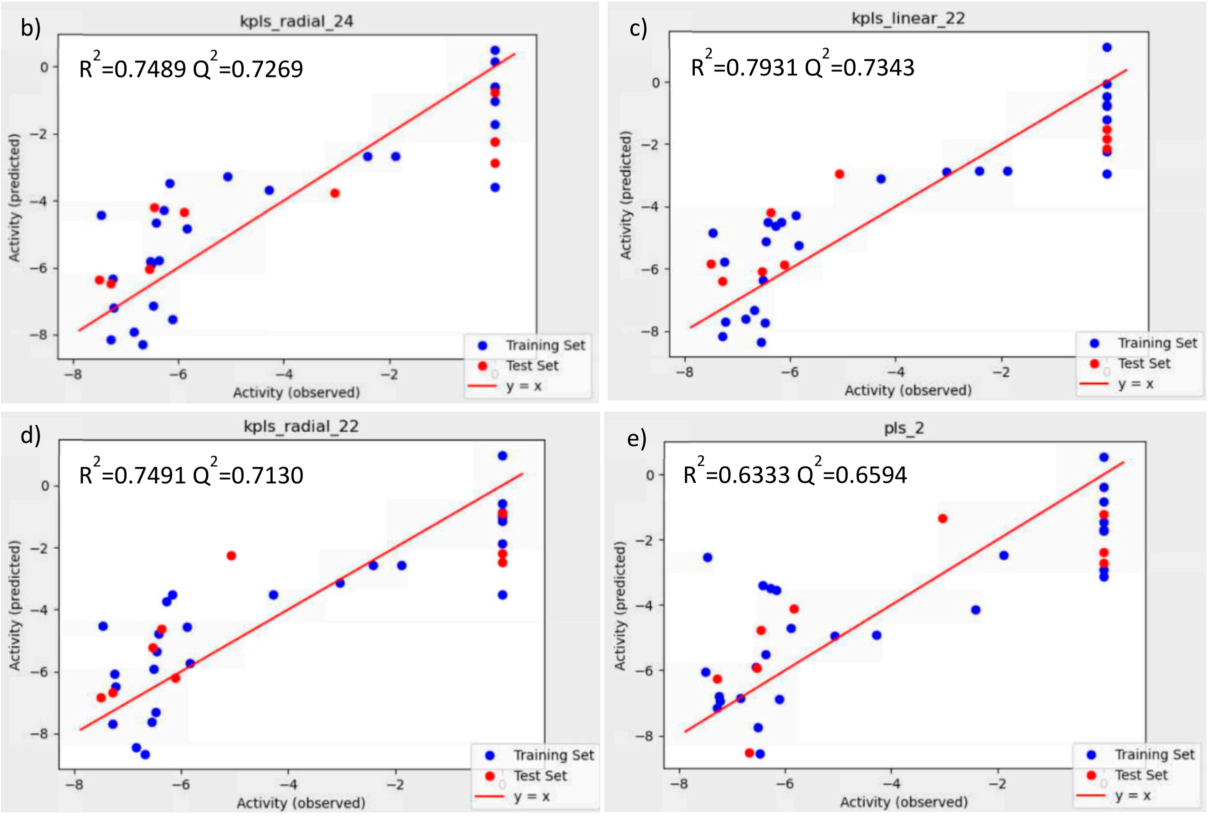

Figure S13. Scatter plot about performance for a) refer to the main paper, b) kpls_radial_24, c) kpls_linear_22, d) kpls_radial_22, and e) pls_2 models of *Plasmopara viticola* without using validation set.

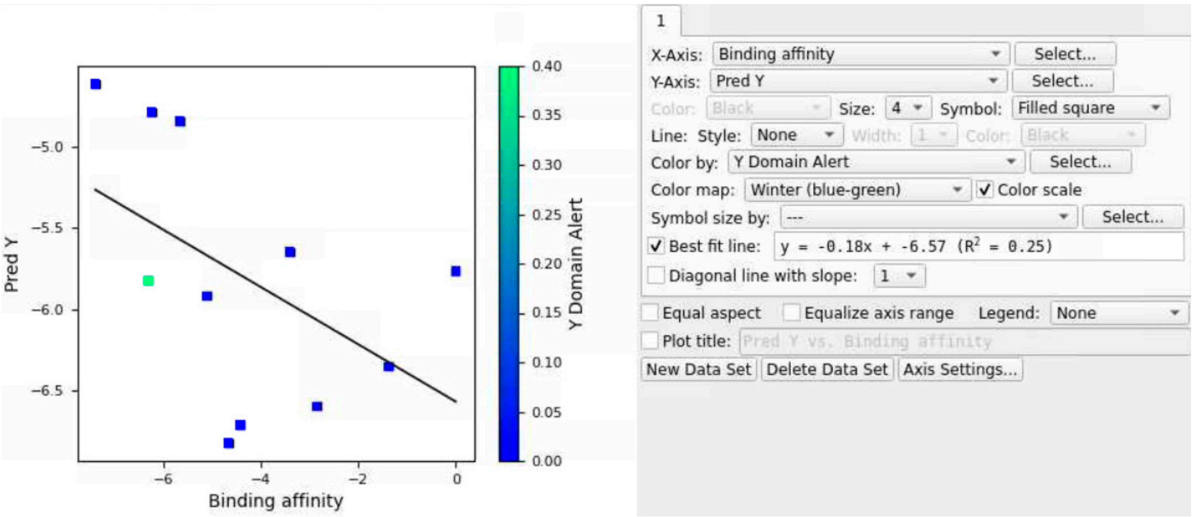

Figure S14. Scatter plot of external validation set for all top five models after removing six outliers in Figure 12 (c).

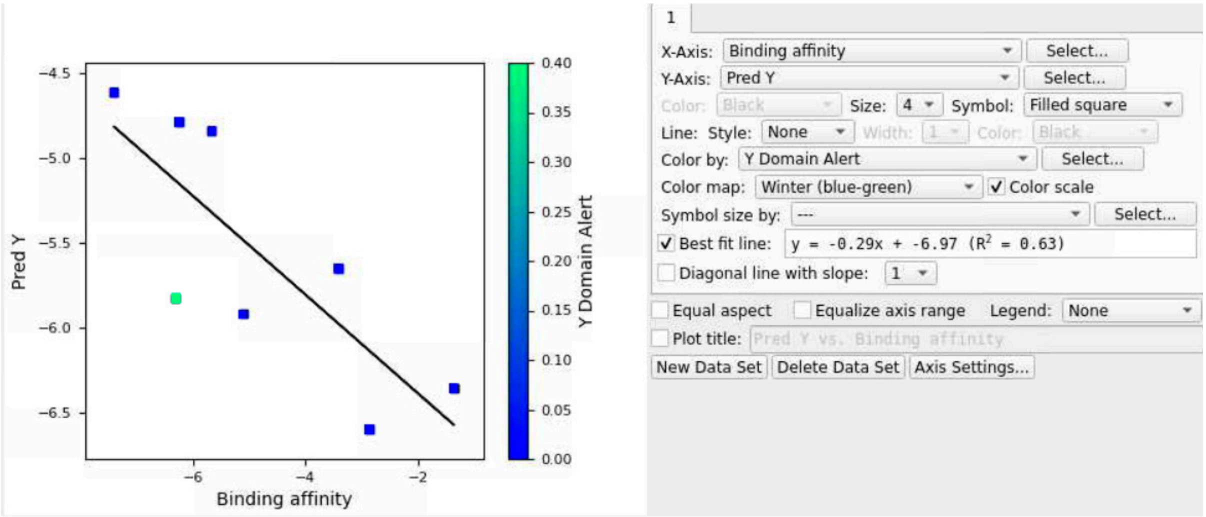

Figure S15. Scatter plot of external validation set for all top five models after removing three outliers in Figure S14.

Model Report

Model Code	Score	S.D.	R^2	RMSE	Q^2	Q^2 MW (Null Hypothesis)
kpls_linear_39	0.7733	1.4315	0.7953	1.4160	0.7624	0.1066
kpls_desc_31	0.7705	1.5857	0.7659	1.4005	0.7614	-0.3689
kpls_dendritic_39	0.7704	1.3903	0.8069	1.4045	0.7662	0.1066
kpls_linear_2	0.7680	1.3868	0.8058	1.4107	0.7885	0.1942
kpls_linear_31	0.7632	1.5812	0.7571	1.4079	0.7589	-0.3689
kpls_dendritic_31	0.7622	1.5533	0.7656	1.4777	0.7344	-0.3689
kpls_radial_31	0.7372	1.6622	0.7316	1.5053	0.7244	-0.3689
pls_31	0.7325	1.7211	0.7242	1.4936	0.7286	-0.3689
kpls_desc_2	0.6927	1.7091	0.7173	1.6569	0.7083	0.1942
pls_2	0.6753	1.8580	0.6658	1.6730	0.7025	0.1942

Figure S16. Top 10-ranked QSAR model reports with validation set for *Plasmopara viticola*.

b)

Training Set		Test Set	
S.D.	R^2	RMSE	Q^2
1.5857	0.7659	1.4005	0.7614

Optimum number of factors = 2

ID	Set	Y(Obs)	Y(Pred)	Error	Name
1	train	0.0000	-0.0223	-0.0223	Azoxystrobin
2	train	-6.1770	-2.8913	3.2857	Coumoxystrobin
3	test	0.0000	0.1754	0.1754	Enoxastrobin
4	train	0.0000	0.7086	0.7086	Fluoxastrobin
5	train	0.0000	0.3846	0.3846	Metyltetraprole
6	train	0.0000	-0.9345	-0.9345	Orysastrobin
7	train	0.0000	-3.2886	-3.2886	Picoxystrobin
8	train	0.0000	-0.9671	-0.9671	Pyraclostrobin
9	train	0.0000	-2.0821	-2.0821	pyrametostrobin
10	train	0.0000	-0.9167	-0.9167	Pyraoxystrobin
11	test	-5.8440	-4.0786	1.7654	Pyribencarb
12	test	0.0000	-2.0481	-2.0481	Triclopyricarb
13	train	0.0000	-1.8613	-1.8613	Captan
14	train	-3.0380	-2.2697	0.7683	Ferbam
15	train	-5.8910	-4.3970	1.4940	Folpet
16	train	-1.8780	-2.6440	-0.7660	Mancozeb
17	train	-4.2850	-4.1806	0.1044	Thiram
18	test	-2.4080	-4.8237	-2.4157	Zineb
19	train	-6.5560	-6.3244	0.2316	Zoxamide
20	train	-6.5160	-8.1444	-1.6284	Sedaxane
21	train	-6.8510	-7.1294	-0.2784	Piperalin
22	train	-6.4860	-8.6045	-2.1185	Inpyrfluxam
23	train	-7.2910	-6.9206	0.3704	Penconazole
24	train	-6.4640	-6.0181	0.4459	Ferimzone
25	train	-7.5040	-7.0410	0.4630	Cyflufenamid
26	test	-6.5290	-6.3066	0.2224	Diclomezine
27	train	-6.4320	-3.8631	2.5689	Dichlobentiazox
28	train	-6.2790	-3.8005	2.4785	Flufenoxystrobin
29	train	-7.2240	-6.7431	0.4809	Azaconazole
30	test	-6.1090	-6.3899	-0.2809	Fenoxanil
31	train	-7.2400	-6.2985	0.9415	Iprodione
32	test	-7.2910	-5.8168	1.4742	Penthiopyrad
33	test	-6.6800	-7.1401	-0.4601	Oxathiapiprolin
34	train	-5.0670	-4.9292	0.1378	Metominostrobin

c)

Training Set		Test Set	
S.D.	R^2	RMSE	Q^2
1.3903	0.8069	1.4045	0.7662

Optimum number of factors = 1

ID	Set	Y(Obs)	Y(Pred)	Error	Name
1	test	0.0000	-2.4017	-2.4017	Azoxystrobin
2	train	-6.1770	-3.8256	2.3514	Coumoxystrobin
3	train	0.0000	0.1218	0.1218	Enoxastrobin
4	train	0.0000	-1.6422	-1.6422	Fluoxastrobin
5	train	0.0000	-0.2450	-0.2450	Metyltetraprole
6	train	0.0000	-1.3003	-1.3003	Orysastrobin
7	train	0.0000	-0.2010	-0.2010	Picoxystrobin
8	train	0.0000	1.3493	1.3493	Pyraclostrobin
9	test	0.0000	-1.5057	-1.5057	pyrametostrobin
10	train	0.0000	0.1005	0.1005	Pyraoxystrobin
11	train	-5.8440	-4.6705	1.1735	Pyribencarb
12	train	0.0000	-0.8057	-0.8057	Triclopyricarb
13	train	0.0000	-3.3248	-3.3248	Captan
14	train	-3.0380	-3.5084	-0.4704	Ferbam
15	train	-5.8910	-4.5728	1.3182	Folpet
16	train	-1.8780	-3.4164	-1.5384	Mancozeb
17	test	-4.2850	-3.6047	0.6803	Thiram
18	train	-2.4080	-3.4164	-1.0084	Zineb
19	train	-6.5560	-7.8953	-1.3393	Zoxamide
20	test	-6.5160	-5.6900	0.8260	Sedaxane
21	train	-6.8510	-7.4287	-0.5777	Piperalin
22	train	-6.4860	-7.5701	-1.0841	Inpyrfluxam
23	test	-7.2910	-6.4012	0.8898	Penconazole
24	train	-6.4640	-5.2502	1.2138	Ferimzone
25	test	-7.5040	-5.1255	2.3785	Cyflufenamid
26	test	-6.5290	-6.1485	0.3805	Diclomezine
27	train	-6.4320	-4.6016	1.8304	Dichlobentiazox
28	train	-6.2790	-4.4093	1.8697	Flufenoxystrobin
29	train	-7.2240	-7.2565	-0.0325	Azaconazole
30	test	-6.1090	-6.0332	0.0758	Fenoxanil
31	train	-7.2400	-6.0561	1.1839	Iprodione
32	train	-7.2910	-7.7458	-0.4548	Penthiopyrad
33	train	-6.6800	-6.8544	-0.1744	Oxathiapiprolin
34	train	-5.0670	-3.3804	1.6866	Metominostrobin

d)

Training Set		Test Set	
S.D.	R^2	RMSE	Q^2
1.3868	0.8058	1.4107	0.7885

Optimum number of factors = 1

ID	Set	Y(Obs)	Y(Pred)	Error	Name
1	test	0.0000	-2.3820	-2.3820	Azoxystrobin
2	train	-6.1770	-3.6406	2.5364	Coumoxystrobin
3	train	0.0000	0.2951	0.2951	Enoxastrobin
4	train	0.0000	-1.3615	-1.3615	Fluoxastrobin
5	train	0.0000	-1.2162	-1.2162	Metyltetraprole
6	train	0.0000	-0.5476	-0.5476	Orysastrobin
7	train	0.0000	-0.2526	-0.2526	Picoxystrobin
8	test	0.0000	-0.4025	-0.4025	Pyraclostrobin
9	train	0.0000	-0.1326	-0.1326	pyrametostrobin
10	train	0.0000	0.6205	0.6205	Pyraoxystrobin
11	train	-5.8440	-4.7601	1.0839	Pyribencarb
12	test	0.0000	-1.8649	-1.8649	Triclopyricarb
13	train	0.0000	-3.3026	-3.3026	Captan
14	test	-3.0380	-3.8695	-0.8315	Ferbam
15	test	-5.8910	-3.6734	2.2176	Folpet
16	train	-1.8780	-3.6541	-1.7761	Mancozeb
17	train	-4.2850	-3.8044	0.4806	Thiram
18	train	-2.4080	-3.6541	-1.2461	Zineb
19	test	-6.5560	-7.0936	-0.5376	Zoxamide
20	test	-6.5160	-5.6914	0.8246	Sedaxane
21	train	-6.8510	-7.1765	-0.3255	Piperalin
22	train	-6.4860	-7.3919	-0.9059	Inpyrfluxam
23	test	-7.2910	-7.1210	0.1700	Penconazole
24	train	-6.4640	-5.0065	1.4575	Ferimzone
25	train	-7.5040	-7.2713	0.2327	Cyflufenamid
26	train	-6.5290	-6.3230	0.2060	Diclomezine
27	train	-6.4320	-4.3922	2.0398	Dichlobentiazox
28	train	-6.2790	-4.3666	1.9124	Flufenoxystrobin
29	train	-7.2240	-7.6193	-0.3953	Azaconazole
30	train	-6.1090	-7.4680	-1.3590	Fenoxanil
31	train	-7.2400	-6.2636	0.9764	Iprodione
32	train	-7.2910	-7.7966	-0.5056	Penthiopyrad
33	train	-6.6800	-7.2093	-0.5293	Oxathiapiprolin
34	train	-5.0670	-3.0523	2.0147	Metominostrobin

e)

Training Set		Test Set	
S.D.	R^2	RMSE	Q^2
1.5812	0.7571	1.4079	0.7589

Optimum number of factors = 1

ID	Set	Y(Obs)	Y(Pred)	Error	Name
1	train	0.0000	-0.7023	-0.7023	Azoxystrobin
2	train	-6.1770	-4.0529	2.1241	Coumoxystrobin
3	test	0.0000	-1.3660	-1.3660	Enoxastrobin
4	train	0.0000	-1.1355	-1.1355	Fluoxastrobin
5	train	0.0000	-0.8193	-0.8193	Metyltetraprole
6	train	0.0000	-1.9540	-1.9540	Orysastrobin
7	train	0.0000	0.0689	0.0689	Picoxystrobin
8	train	0.0000	0.5797	0.5797	Pyraclostrobin
9	train	0.0000	-0.4633	-0.4633	pyrametostrobin
10	train	0.0000	0.3089	0.3089	Pyraoxystrobin
11	test	-5.8440	-3.7030	2.1410	Pyribencarb
12	test	0.0000	-1.7012	-1.7012	Triclopyricarb
13	train	0.0000	-3.9677	-3.9677	Captan
14	train	-3.0380	-3.9369	-0.8989	Ferbam
15	train	-5.8910	-4.5429	1.3481	Folpet
16	train	-1.8780	-4.1376	-2.2596	Mancozeb
17	train	-4.2850	-4.1001	0.1849	Thiram
18	test	-2.4080	-4.1376	-1.7296	Zineb
19	train	-6.5560	-7.6134	-1.0574	Zoxamide
20	train	-6.5160	-6.3305	0.1855	Sedaxane
21	train	-6.8510	-7.2062	-0.3552	Piperalin
22	train	-6.4860	-6.9609	-0.4749	Inpyrfluxam
23	train	-7.2910	-8.1309	-0.8399	Penconazole
24	train	-6.4640	-4.2480	2.2160	Ferimzone
25	train	-7.5040	-7.3227	0.1813	Cyflufenamid
26	test	-6.5290	-5.7559	0.7731	Diclomezine
27	train	-6.4320	-4.0391	2.3929	Dichlobentiazox
28	train	-6.2790	-3.9048	2.3742	Flufenoxystrobin
29	train	-7.2240	-7.6264	-0.4024	Azaconazole
30	test	-6.1090	-6.2949	-0.1859	Fenoxanil
31	train	-7.2400	-6.1569	1.0831	Iprodione
32	test	-7.2910	-6.3324	0.9586	Penthiopyrad
33	test	-6.6800	-5.2757	1.4043	Oxathiapiprolin
34	train	-5.0670	-2.7843	2.2827	Metominostrobin

Figure S17. Model reports for a) refer to the main paper, b) kpls_desc_31, c) kpls_dendritic_39, d) kpls_linear_2, and e) kpls_linear_31 models of *Plasmopara viticola* using validation set.

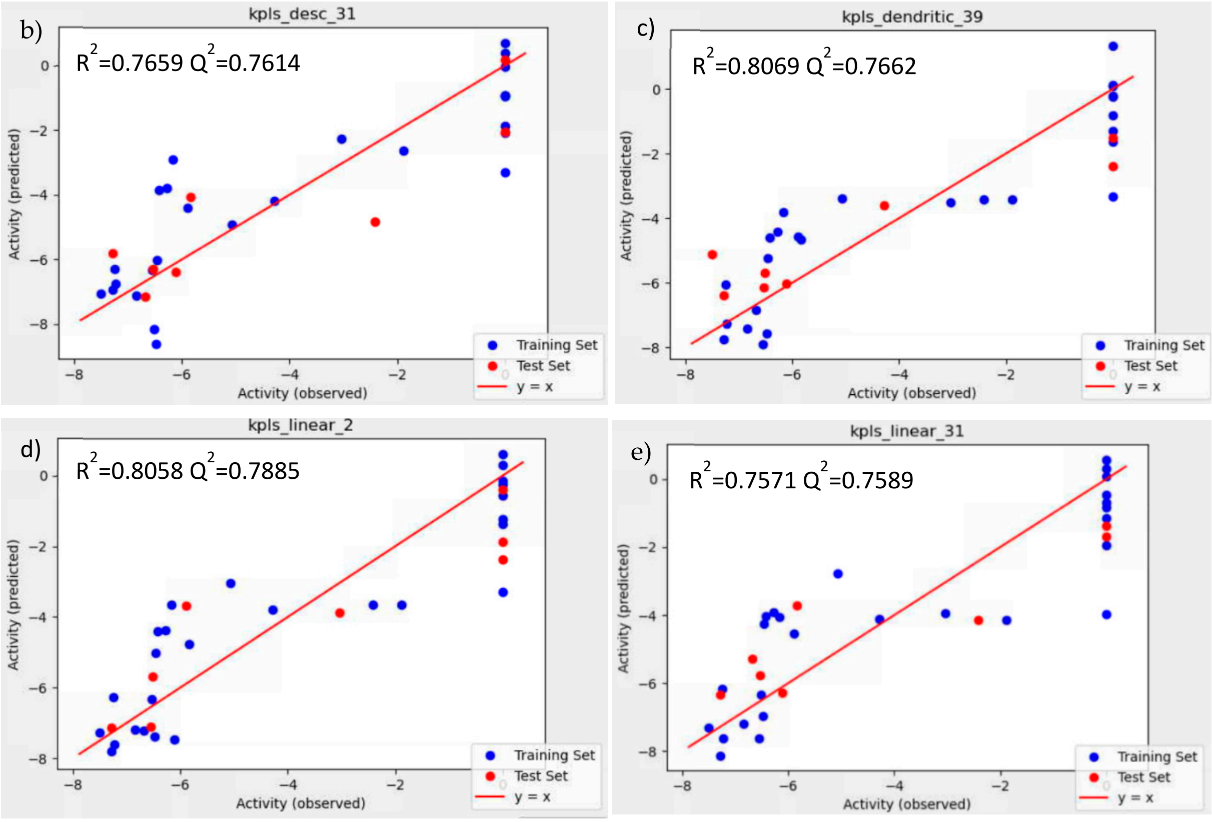

Figure S18. Scatter plot about performance for a) refer to the main paper, b) kpls_desc_31, c) kpls_dendritic_39, d) kpls_linear_2, and e) kpls_linear_31 models of *Plasmopara viticola* using validation set.

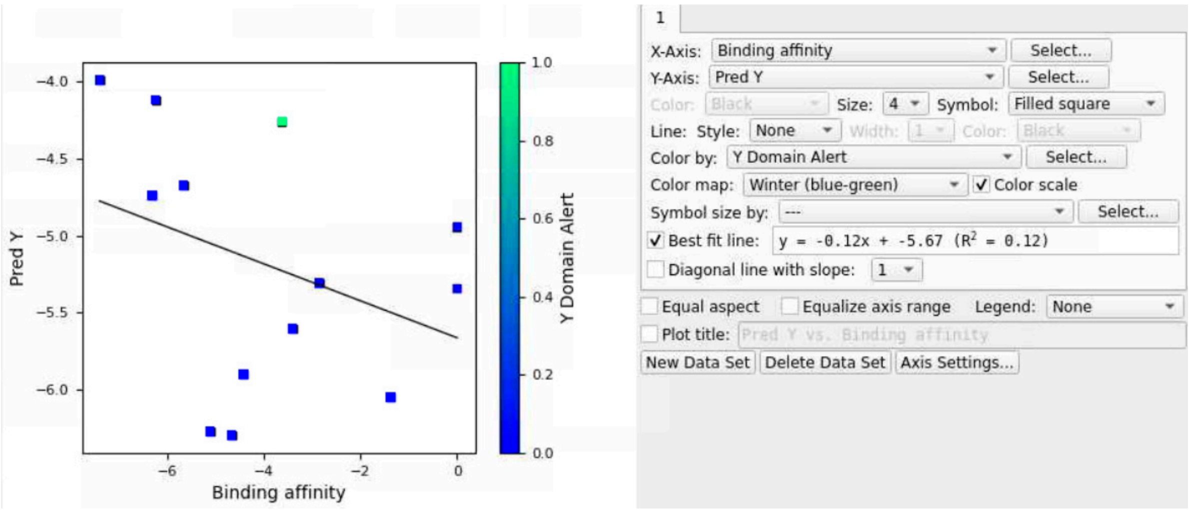

Figure S19. Scatter plot of external validation set after removing four outliers in Figure 13 (c).

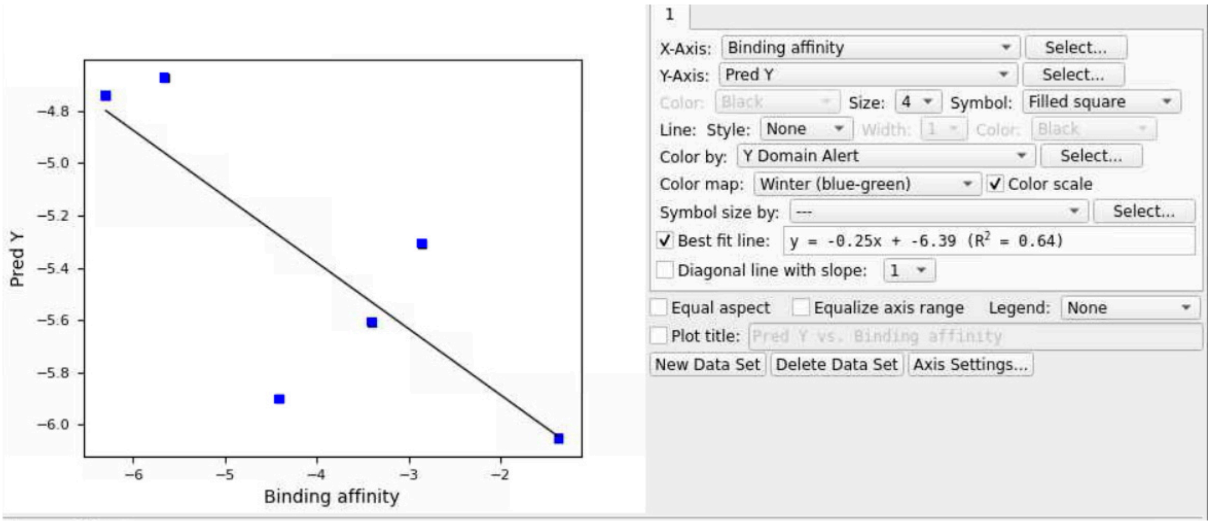

Figure S20. Scatter plot of external validation set after removing seven outliers in Figure S19.
